# Supplementary figures and images for: Dependence of diffusion in Escherichia coli cytoplasm on protein size, environmental conditions, and cell growth
Source: eLife. 2022 Dec 5;11:e82654. doi: 10.7554/eLife.82654 (PMC9810338; doi:10.7554/eLife.82654)

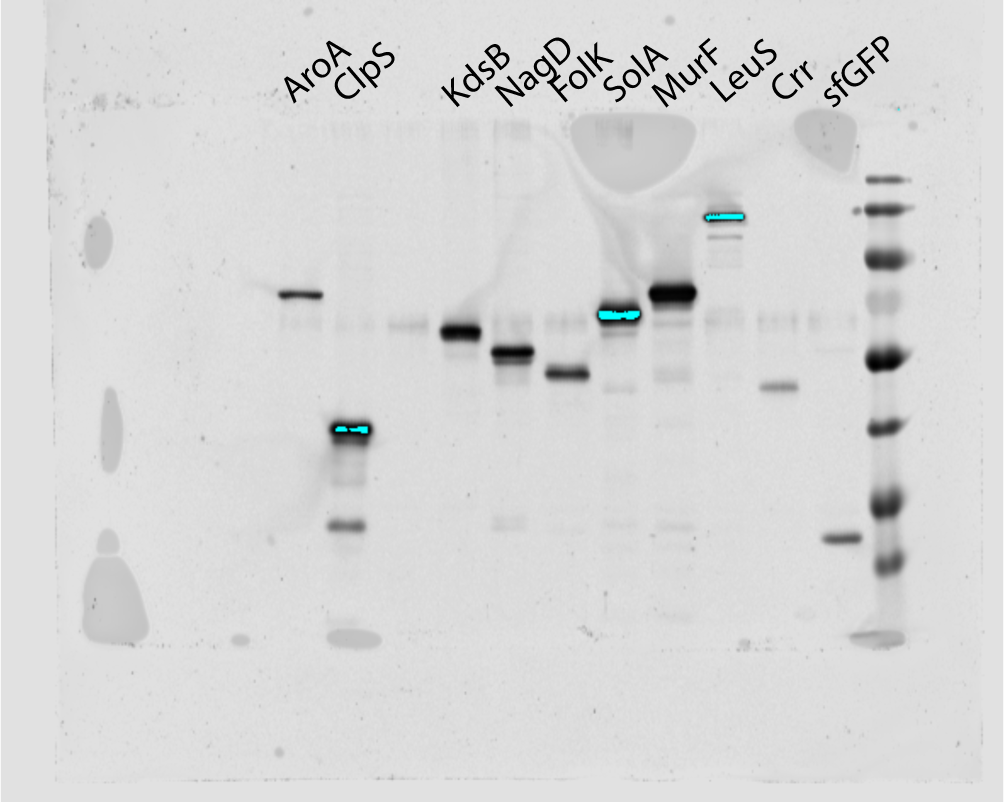

Supplement: Figure 1—figure supplement 1—source data 1. [file elife-82654-fig1-figsupp1-data1.zip › Figure 1 - figure supplement 1 - source data 1.tif]

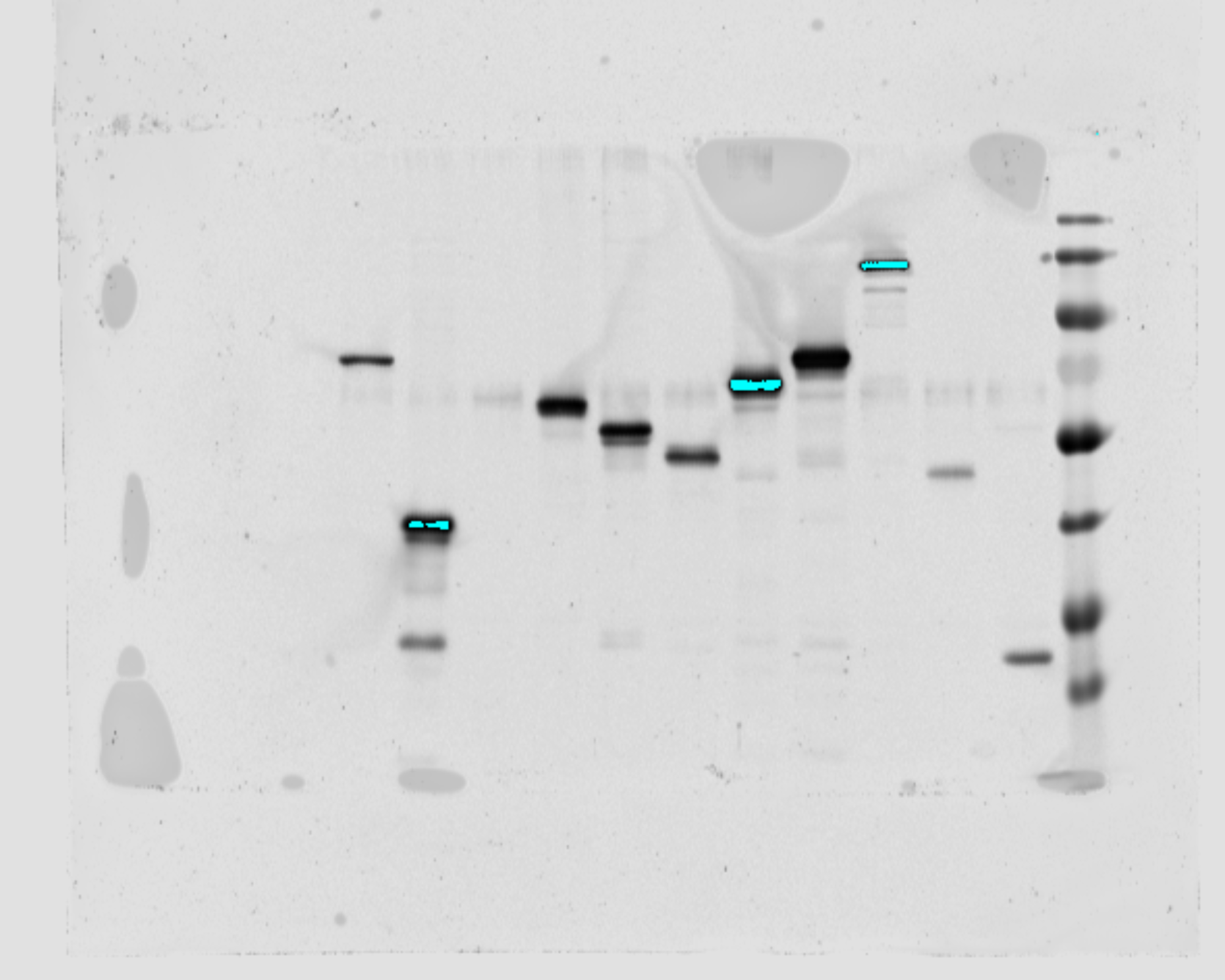

Supplement: Figure 1—figure supplement 1—source data 1. [file elife-82654-fig1-figsupp1-data1.zip › Figure 1 - figure supplement 1 - source data 1__unedited.tif]

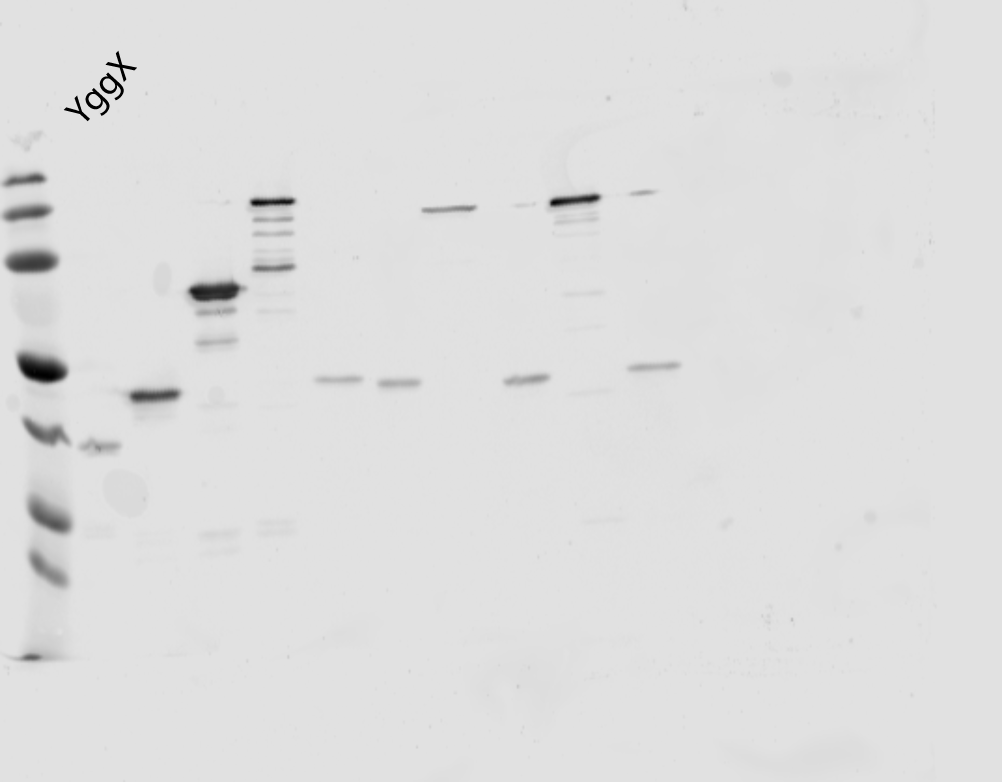

Supplement: Figure 1—figure supplement 1—source data 1. [file elife-82654-fig1-figsupp1-data1.zip › Figure 1 - figure supplement 1 - source data 2.tif]

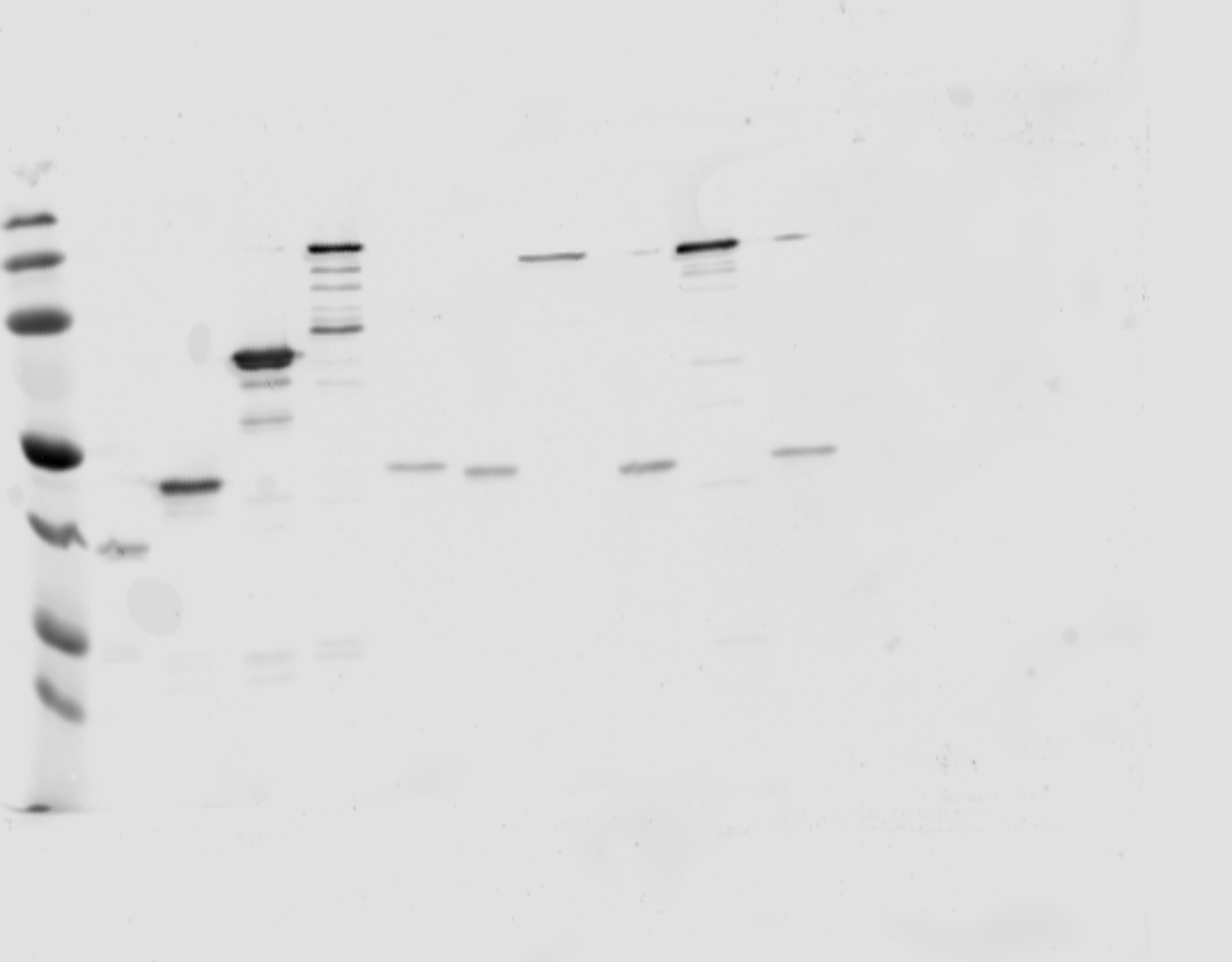

Supplement: Figure 1—figure supplement 1—source data 1. [file elife-82654-fig1-figsupp1-data1.zip › Figure 1 - figure supplement 1 - source data 2_unedited.tif]

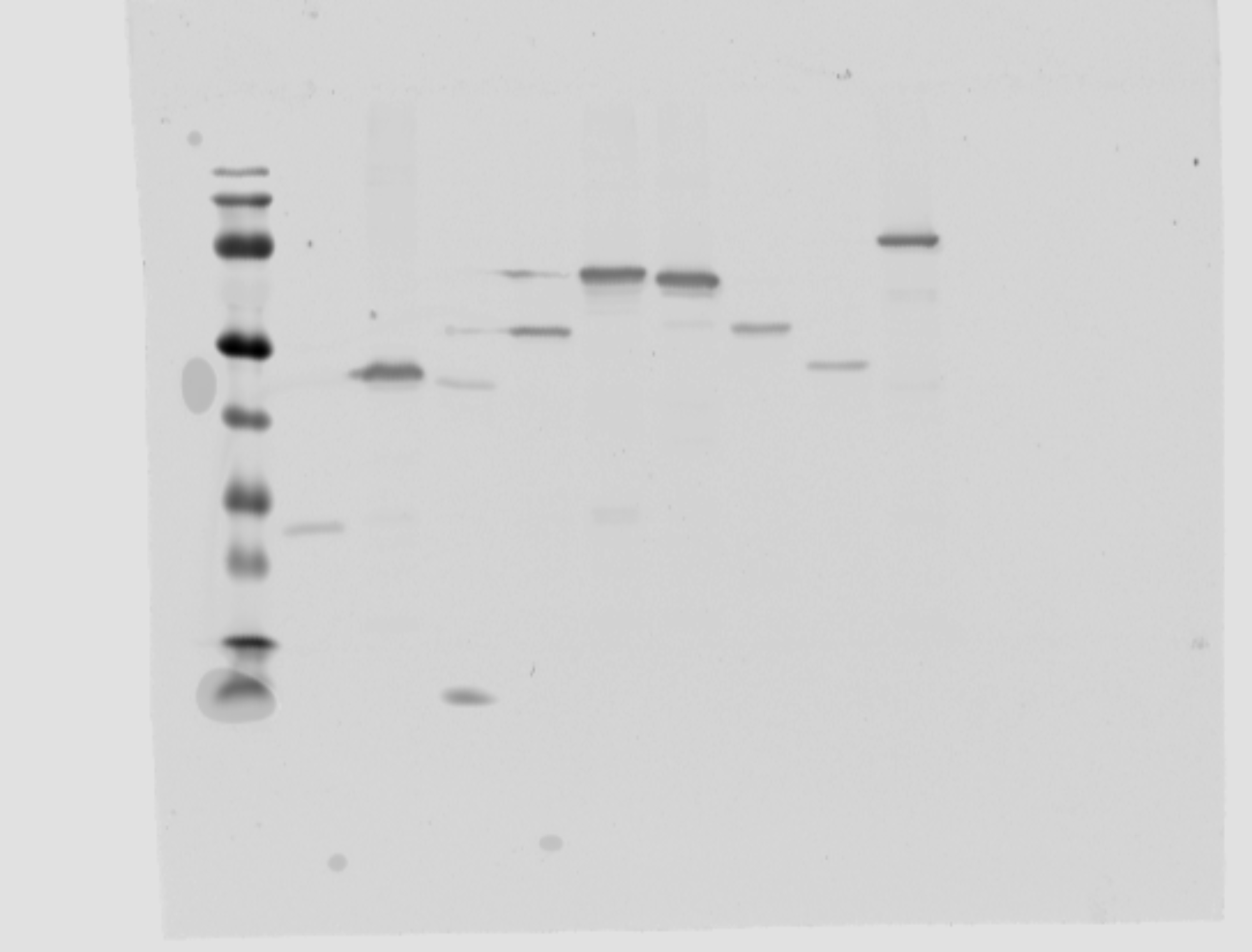

Supplement: Figure 1—figure supplement 1—source data 1. [file elife-82654-fig1-figsupp1-data1.zip › Figure 1 - figure supplement 1 - source data 3_unedited.tif]

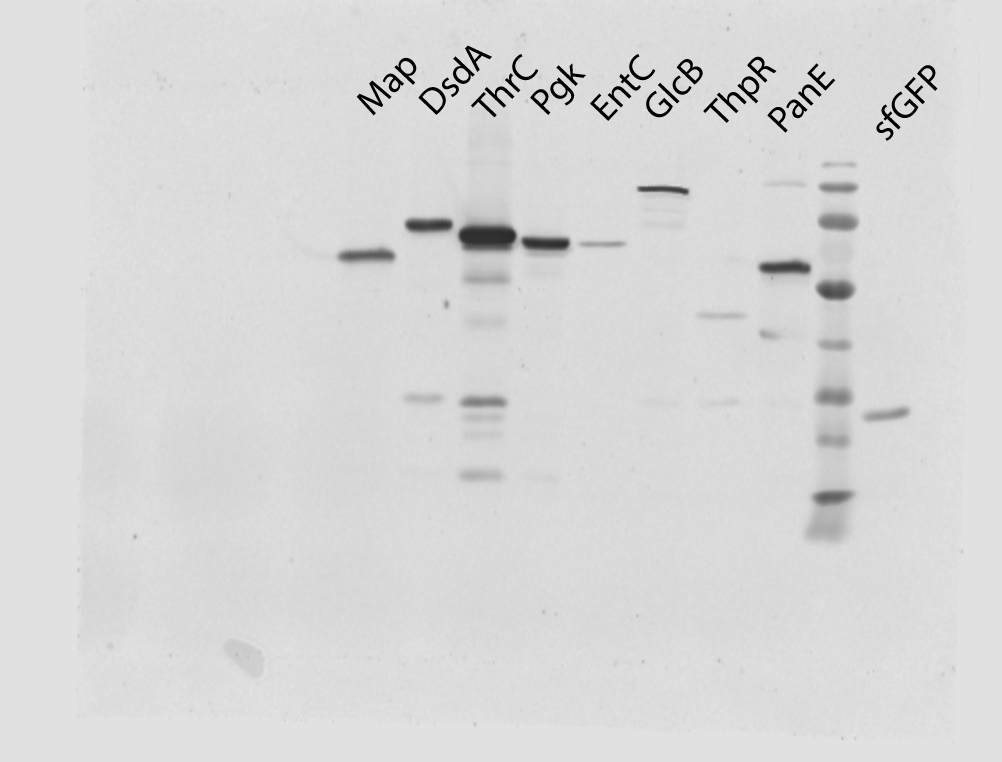

Supplement: Figure 1—figure supplement 1—source data 1. [file elife-82654-fig1-figsupp1-data1.zip › Figure 1 - figure supplement 1 - source data 4.tif]

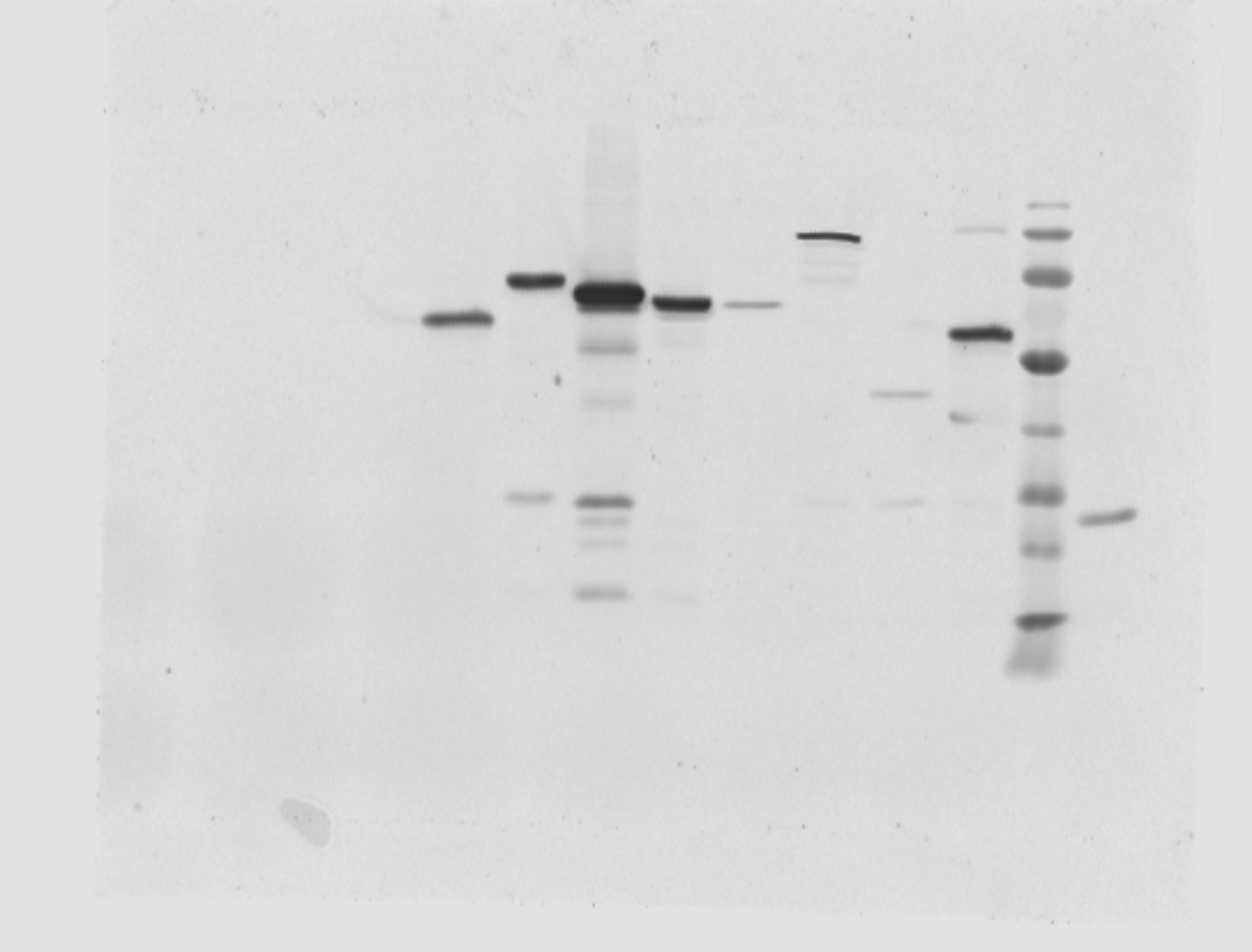

Supplement: Figure 1—figure supplement 1—source data 1. [file elife-82654-fig1-figsupp1-data1.zip › Figure 1 - figure supplement 1 - source data 4_unedited.tif]

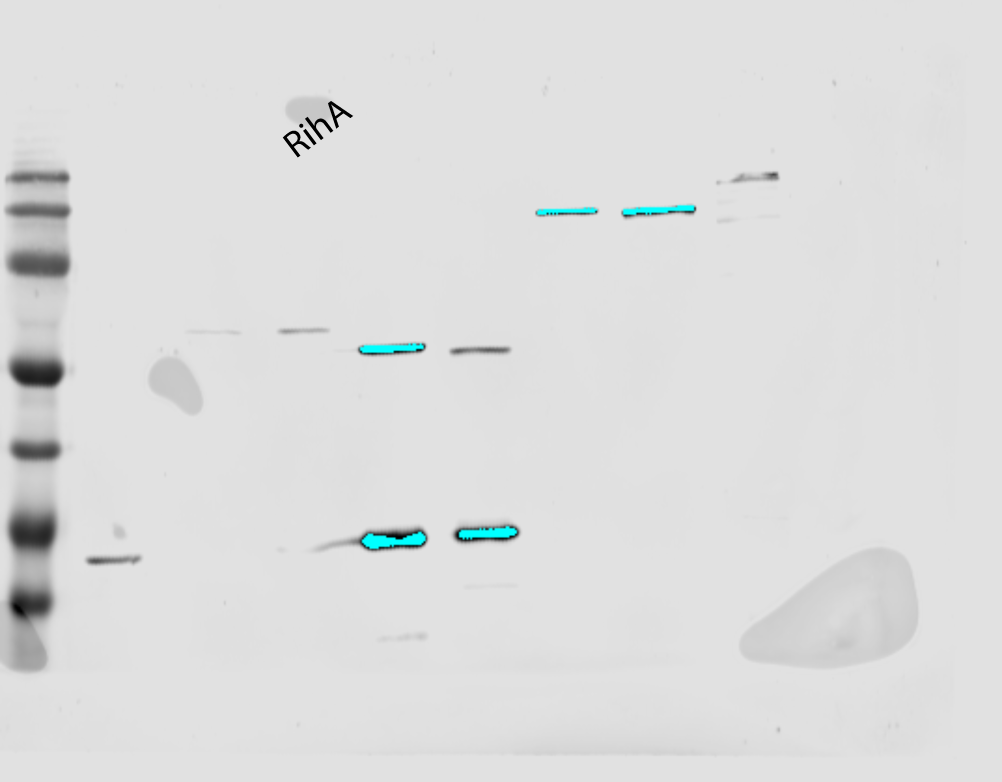

Supplement: Figure 1—figure supplement 1—source data 1. [file elife-82654-fig1-figsupp1-data1.zip › Figure 1 - figure supplement 1 - source data 5.tif]

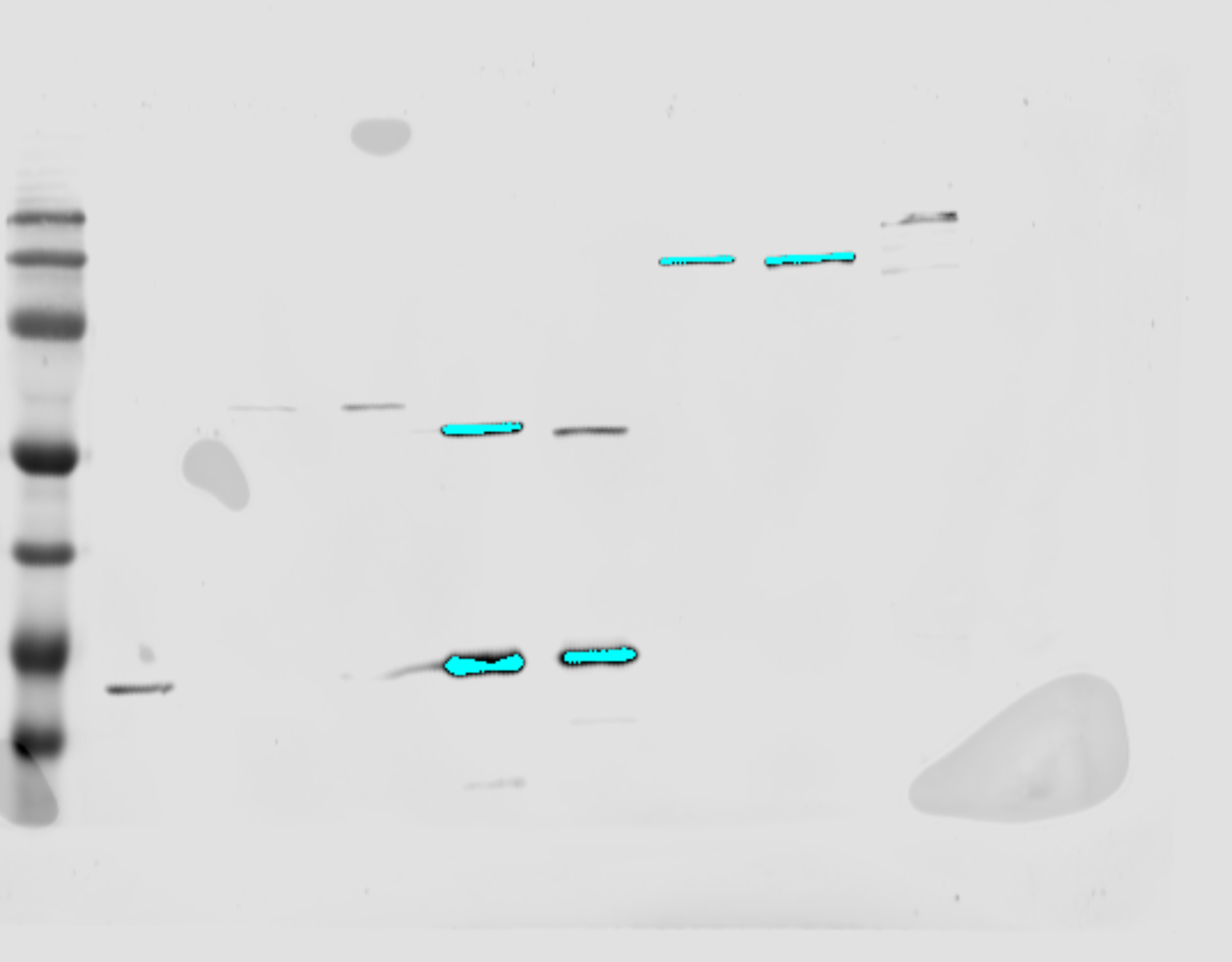

Supplement: Figure 1—figure supplement 1—source data 1. [file elife-82654-fig1-figsupp1-data1.zip › Figure 1 - figure supplement 1 - source data 5_unedited.tif]

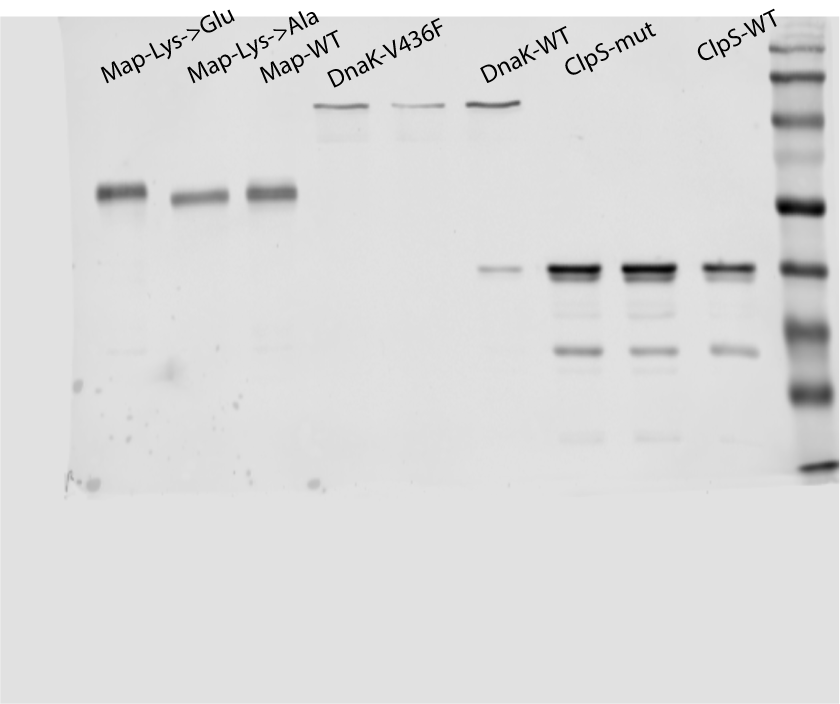

Supplement: Figure 1—figure supplement 9—source data 1. [file elife-82654-fig1-figsupp9-data1.zip › Figure 1 - figure supplement 9 source data 1.tif]

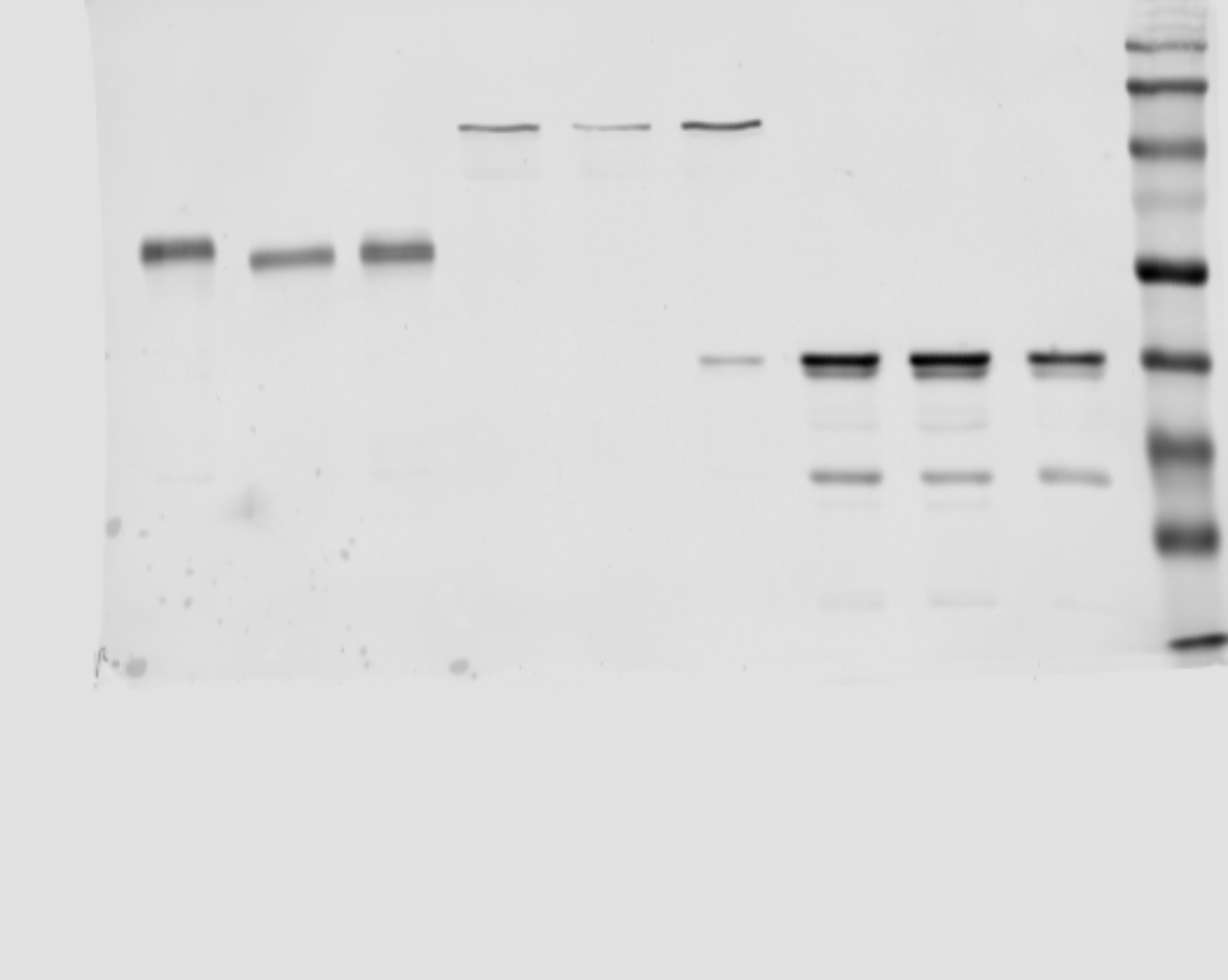

Supplement: Figure 1—figure supplement 9—source data 1. [file elife-82654-fig1-figsupp9-data1.zip › Figure 1 - figure supplement 9 source data 1_unedited.tif]
